# Synthesis of the aglycon of scorzodihydrostilbenes B and D

**DOI:** 10.3762/bjoc.15.56

**Published:** 2019-03-06

**Authors:** Katja Weimann, Manfred Braun

**Affiliations:** 1 Institute of Organic and Macromolecular Chemistry, Heinrich-Heine-University Düsseldorf, Universitätsstr. 1, D-40225 Düsseldorf, Germany

**Keywords:** C–H activation, hydroarylation, phenols, regioselectivity, ruthenium

## Abstract

Benzyl- and methyl-protected 2,4-dihydroxyacetophenones are added under ruthenium catalysis to 4-methoxy- and 3,4-dimethoxystyrene in a completely regioselective manner. Thus, oxygenated dihydrostilbenes are obtained that feature the skeleton of scorzodihydrostilbenes – antioxidative agents that were recently isolated from *Scorzonera radiata*. Selective deprotection liberates the corresponding phenols, among them the aglycon of scorzodihydrostilbenes B and D.

## Introduction

**Scheme 1:**
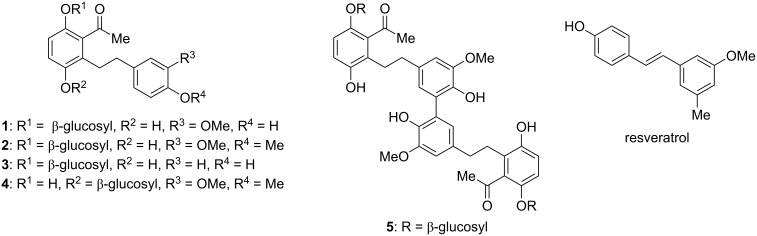
Structures of scorzodihydrostilbenes A–E (**1**–**5**) and resveratrol.

Among phytochemicals with strong allelopathic effects, various natural products are found that feature the structural motif of dihydrostilbene with multiple phenolic functionality. These natural products that form as secondary metabolites on a branch of flavonoid biosynthesis found wide interest for their various biological effects like anti-oxidative and biofouling-preventing activity [1]. From crude extracts of the Mongolian medicinal plant *Scorzonera radiata*, that is used in folk medicine for the treatment of poisonous ulcers, fever, and various other diseases [2–4], five new dihydrostilbenes, named scorzodihydrostilbenes A–E (**1**–**5**), were isolated in 2009 and their structures were determined (Scheme 1) [5]. They all are β-glucosides with highly oxygenated aryl rings. Scorzodihydrostilbene E (**5**) features a dimeric skeleton that originates from an oxidative coupling of compound **1**. The natural products exhibited antioxidative activity that was partly stronger than that of the well-known naturally occurring antioxidant resveratrol [5]. In this article, we describe a synthetic approach that takes advantage of a regioselective, ruthenium-catalyzed C–H activation [6] and makes accessible not only the skeleton of dihydrostilbenes with multiple phenolic ether functionality but also the aglycon of scorzodihydrostilbenes B and D (**2** and **4**, R^1^ = R^2^ = H, instead of β-glycosyl, respectively).

## Results and Discussion

Most syntheses of dihydrostilbenes rely on a carbonyl olefination followed by hydrogenation of the stilbene [7–9]. However, this route appeared not attractive, particularly as our attempts to hydrogenate highly substituted stilbenes that would serve as the precursors of scorzodihydrostilbenes had failed, presumably due to steric hindrance caused by the accumulation of substituents at the acetophenone moiety [10]. We felt that a straightforward access to the scorzodihydrostilbene aglycons would be possible by using the transition metal-mediated, regioselective, chelation-directed activation of an aryl–hydrogen bond. A promising approach was seen in an application of Murai’s elegant ruthenium-catalyzed *ortho*-functionalization of aryl alkyl ketones and their addition to olefins [11–15]. This route appeared particularly attractive as it leads in an atom-economic manner directly to the carbon skeleton of scorzodihydrostilbenes, starting from suitably substituted acetophenone and styrene derivatives. Furthermore, Murai’s protocol offers another advantage: the regioselective formation of the anti-Markovnikov product. Here, a modified version based upon an in situ generation of the ruthenium complex described by Genet and Darses [16] was applied.

**Scheme 2:**
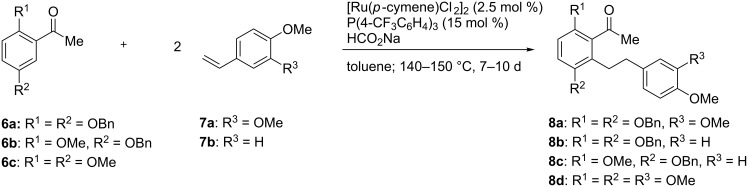
Synthesis of dihydrostilbenes **8a–d** by ruthenium-catalyzed addition of ketones **6** to styrenes **7**. Yields: **8a**: 65%; **8b**: 68%; **8c**: 73%; **8d**: 40%.

Thus, O-protected 2,5-dihydroxyacetophenones **6** were submitted to a hydroarylation reaction with two equivalents of styrenes **7** in the presence of [Ru(*p*-cymene)Cl_2_]_2_ and P(4-CF_3_C_6_H_4_)_3_. The reactions were performed with sodium formate in toluene in a sealed tube at 140–150 °C. Reaction times of 7 to 10 days turned out to be necessary. Thus, dihydrostilbenes **8a–c** were obtained in fair yields in a single step, whereas in the case of the tetramethoxy-substituted derivative **8d**, the yield was lower (Scheme 2).

**Scheme 3:**
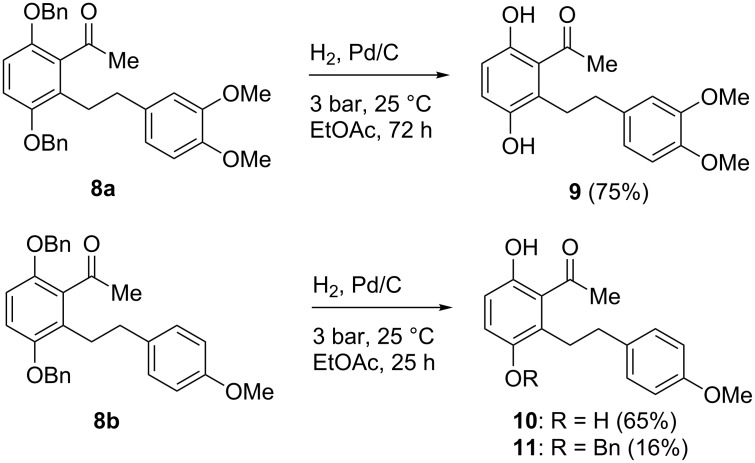
Cleavage of benzyl protecting groups in ketones **8a** and **8b**. Synthesis of scorzodihydrostilbene aglycon **9**.

In order to bring about the cleavage of the benzyl protecting groups in the ketones **8a** and **8b** by hydrogenolysis [17–18], various metal catalysts were tested. It turned out that palladium on charcoal was the only catalytic system that provided satisfying results. Ethyl acetate was found to be the appropriate solvent, whereas the starting materials did not dissolve in methanol or ethanol. The reaction required a hydrogen overpressure of 3 bar for one to three days at room temperature, but even then, the deprotection was not completed in all cases. On the other hand, partial hydrogenation of the aromatic rings had to be suppressed. Nevertheless, the aglycon **9** of scorzodihydrostilbenes B and D (**2** and **4**) was obtained in good yield from **8a**. Hydrogenolysis of ketone **8b** led to hydroquinone **10**, however, along with a minor amount of mono-deprotected phenol **11**. The main product **10** was isolated in pure form by column chromatography, whereas the fraction containing the phenol **11** was still contaminated with hydroquinone **10** (Scheme 3).

**Scheme 4:**
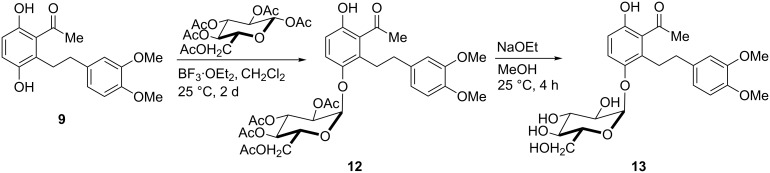
Synthesis of glycoside **12** and deprotected *epi*-scorzodihydrostilbene D (**13**). Yields of isolated products: **12**: 14%; **13**: 17%.

Finally, the glycosylation of the aglycon **9** was briefly studied using Helferich’s method [19]. It turned out that, upon treatment of **9** with β-D-glucose pentaacetate in the presence of an excess of boron trifluoride etherate [20], a regioselective reaction occurred at the phenolic group in *ortho*-position to the alkyl side chain to give mono-glycosylated product **12**. Chelation of the carbonyl and the neighboring phenolic group by the Lewis acid is assumed to be responsible for this regiochemical outcome. The ^1^H NMR spectrum of **12** clearly indicates – by the low-field shift of the phenolic hydrogen chelated with the carbonyl group – that glycosylation had not occurred at this position. Unfortunately, however, the α-anomer **12** formed exclusively, so that, after cleavage of the ester groups, *epi*-scorzodihydrostilbene D (**13**) was obtained as single stereoisomer (Scheme 4).

Obviously, the conditions required in the glycosylation reaction – excess of the Lewis acid and elongated reaction time – resulted in the formation of the thermodynamically favored α-anomer (for details, see Experimental Part and Supporting Information File 1). Attempts aimed at synthesizing the natural β-anomeric scorzodihydrostilbenes A–D are pursued.

## Conclusion

In summary, a straightforward procedure for the synthesis of dihydrostilbenes with multiple phenolic substitutions was opened based upon Murai’s ruthenium-catalyzed hydroarylation of olefins. Thus, the aglycon of scorzodihydrostilbene D became accessible in two steps from readily available starting materials.

## Experimental

**General**: Melting points (uncorrected) were determined with a Büchi 540 melting point apparatus. NMR spectra were recorded with Bruker DXR 600 and DXR 300 spectrometers. Mass spectra were recorded on ion-trap API mass spectrometer Finnigan LCQ Deca (ESI), triple-quadrupole-mass spectrometer Finnigan TSQ 7000, and sector field mass spectrometer Finnigan MAT 8200 (EI, 70 eV), Thermo Finnigan TraceGC Ultra (GC–MS). Column chromatography was performed with Fluka silica gel 60 (230–400 mesh) and thin-layer chromatography was carried out by using Merck TLC Silicagel 60F254 aluminium sheets. Tetrahydrofuran (THF) and toluene were refluxed under nitrogen over sodium wire and a small amount of benzophenone until the dark blue color of the solution persisted. Then, the solvents were distilled under nitrogen and taken from the distillation flask, which was closed by a septum, by syringes or cannulas. 2,5-Dimethoxyacetophenone (**6c**) and 4-methoxystyrene (**7b**) were purchased from Sigma-Aldrich. 2,5-Dibenzyloxyacetophenone (**6a**) was prepared from commercially available 2,5-dihydroxyacetophenone according to [21].

**1-(5-Benzyloxy-2-methoxyphenyl)ethan-1-one** (**6b**): A mixture of commercially available 5-hydroxy-2-methoxyacetophenone (1.0 g, 6.0 mmol), K_2_CO_3_ (1.24 g, 9.0 mmol), benzyl bromide (2.05 g, 12.0 mmol) and DMF (14 mL) was stirred at 25 °C for 5 h. After acidification with 5% hydrochloric acid, the mixture was extracted with three 15 mL portions of ethyl acetate. The combined organic layers were washed with brine, the solvent was removed in a rotary evaporator and the residue was purified by column chromatography (silica gel; ethyl acetate/*n*-hexane, 1:9) to give white, crystalline product **6b** in 99% yield (1.518 g). The spectroscopic data agree with those described in the literature [21].

**1,2-Dimethoxy-4-vinylbenzene** (**7a**): A 100 mL two-necked flask was equipped with a reflux condenser, a magnetic stirrer and a connection to a combined nitrogen-vacuum line, charged with sodium hydride (1.6 g; 60% in mineral oil, 40 mmol) and closed with a septum. *n*-Hexane (20 mL) was injected by syringe and the suspension was stirred for 10 min. Then, stirring was interrupted and the supernatant liquid was removed by syringe. THF (30 mL) and 3,4-dimethoxybenzaldehyde (3.32 g, 20.0 mmol), and methyltriphenylphosphonium bromide (10 g, 28 mmol) were added and the mixture was refluxed for 24 h. After cooling in an ice bath, deionized water (5 mL) was added and the mixture was extracted with three 30 mL portions of ethyl acetate. The combined organic layers were washed with brine, dried with sodium sulfate and concentrated under reduced pressure. The crude product was purified by column chromatography (ethyl acetate/*n*-hexane, 1:9) to give 2.01 g (61%) of styrene **7a** as a colorless liquid. The spectroscopic data agree with those described in the literature [22].

**General procedure for the synthesis of dihydrostilbenes 8**: In a glove-box under argon atmosphere, a 20 mL overpressure tube was charged with ketone **6** (4.0 mmol), styrene **7** (8.0 mmol), [Ru(*p*-cymene)Cl_2_]_2_ (62.0 mg, 0.1 mmol), P(4-CF_3_C_6_H_4_)_3_ (190 mg, 0.6 mmol), and sodium formate (82 mg; 1.2 mmol). Freshly distilled toluene (3 mL) was added, and the mixture was kept in the sealed tube for 7 to 10 days at 140 to 150 °C. After cooling to 25 °C, deionized water (2 mL) was added and the mixture was extracted with five 20 mL portions of ethyl acetate. The combined organic layers were washed with brine and dried with MgSO_4_. The solvent was removed in a rotary evaporator and the residue was purified by column chromatography. According to this procedure, the following compounds were obtained:

**1-[3,6-Bis(benzyloxy)-2-(3,4-dimethoxyphenethyl)phenyl)]ethan-1-one (8a):** Prepared from 2,4-bis(benzyloxy)acetophenone (**6a**, 760 mg, 2.3 mmol) and 3,4-dimethoxystyrene (**7a**, 860 mg, 5.2 mmol) in 2 mL of toluene at 150 °C for 7 d. The crude product was purified by column chromatography (silica gel; ethyl acetate/*n*-hexane, 1:7) and **8a** was obtained as a greenish syrup in 65% yield (741 mg). *R*_f_ 0.1; ^1^H NMR (CDCl_3_, 600 MHz) δ 2.40 (s, 3H), 2.80 (s, 4H), 3.73 (s, 3H), 3.84 (s, 3H), 5.03 (s, 2H), 5.04 (s, 2H), 6.60 (d, *J* = 1.8 Hz, 1H), 6.67–6.69 (dd, *J* = 8.1, 2.0 Hz, 1H), 6.74 (d, *J* = 8.1 Hz, 1H), 6.77 (d, *J* = 8.9 Hz, 1H), 6.85 (d, *J* = 8.9 Hz, 1H), 7.31–7.41 (m, 8H), 7.45 (m, 2H); ^13^C NMR (CDCl_3_, 150 MHz) δ 30.8, 32.6, 36.3, 55.9, 56.1, 70.3, 70.1, 110.8, 111.3, 112.0, 112.7, 120.5, 127.3, 127.3, 127.5, 127.5, 128.1, 128.1, 128.5, 128.7, 128.7, 133.7, 135.2, 137.0, 137.4, 147.3, 148.9, 149.2, 151.5, 205.4; HRMS (ESI): [M + H]^+^ calcd for C_32_H_33_O_5_, 497.2328; found, 497.2321.

**1-[3,6-Bis(benzyloxy)-2-(4-methoxyphenethyl)phenyl]ethan-1-one (8b):** Prepared from 2,5-bis(benzyloxy)acetophenone (**6a**, 665 mg, 2.0 mmol) and commercially available 4-methoxystyrene (**7b**, 537 mg, 4.0 mmol) in 1.5 mL of toluene at 145 °C for 10 d. The crude product was purified by column chromatography (silica gel; ethyl acetate/*n*-hexane, 1:18) and **8b** was obtained as a yellowish syrup in 68% yield (633 mg); *R*_f_ 0.4; ^1^H NMR (CDCl_3_, 600 MHz) δ 2.40 (s, 3H), 2.80 (s, 4H), 3.73 (s, 3H), 5.03 (s, 2H), 5.04 (s, 2H), 6.60 (s, 1H), 6.67–6.69 (m, 1H), 6.73–6.78 (m, 2H), 6.85 (d, *J* = 8.9 Hz, 1H), 7.31–7.41 (m, 8H), 7.45 (m, 2H); ^13^C NMR (CDCl_3_, 150 MHz) δ 30.8, 32.6, 36.3, 55.9, 70.3, 70.1, 110.8, 111.3, 112.0, 112.7, 120.5, 127.3, 127.3, 127.5, 127.5, 128.1, 128.1, 128.5, 128.7, 128.7, 128.7, 128.7, 133.7, 135.2, 137.0, 137.4, 147.3, 148.9, 149.2, 151.5, 205.4; HRMS (ESI): [M + H]^+^ calcd for C_31_H_31_O_4_, 467.2222; found, 467.2216.

**1-[3-(Benzyloxy)-6-methoxy-2-(4-methoxyphenethyl)phenyl]ethan-1-one (8c):** Prepared from 5-(benzyloxy)-2-methoxyacetophenone (**6b**, 513 mg, 2.0 mmol) and commercially available 4-methoxystyrene (**7b**, 537 mg, 4.0 mmol). The crude product was purified by column chromatography (silica gel; ethyl acetate/*n*-hexane, 1:18) and **8c** was obtained as a greenish syrup in 73% yield (571 mg); *R*_f_ 0.3; ^1^H NMR (CDCl_3_, 600 MHz) δ 2.39 (s, 3H), 2.79 (m, 4H), 3.697 (s, 3H), 3.698 (s, 3H), 5.06 (s, 2H), 6.73 (d, *J* = 8.5 Hz, 1H), 6.78 (d, *J* = 8.5 Hz, 2H), 6.88 (d, *J* = 8.5 Hz, 1H), 7.03 (d, *J* = 8.3 Hz, 2H), 7.35 (t, *J* = 7.3 Hz, 1H), 7.41 (t, *J* = 7.4 Hz, 2H), 7.45 (d, *J* = 7.1 Hz, 2H); ^13^C NMR (CDCl_3_, 150 MHz) δ 30.8, 32.5, 35.9, 55.4, 56.1, 70.8, 109.1, 112.8, 113.9, 113.9, 127.3, 127.3, 128.0, 128.5, 128.7, 128.7, 129.6, 133.1, 134.7, 137.5, 150.1, 151.2, 158.0, 205.5; HRMS (ESI): [M + H]^+^ calcd for C_25_H_27_O_4_, 391.1909; found, 391.1905.

**1-[2-(3,4-Dimethoxyphenethyl)-3,6-dimethoxyphenyl]ethan-1-one (8d):** Prepared from 2,5-dimethoxyacetophenone (**6c**, 168 mg, 1.04 mmol) and 3,4-dimethoxystyrene (**7a**, 348 mg, 2.0 mmol) in 1 mL of toluene at 145 °C for 7 d. The crude product was purified by column chromatography (silica gel; ethyl acetate/*n*-hexane, 1:9) and **8d** was obtained as an orange syrup in 40% yield (145 mg); *R*_f_ 0.35; ^1^H NMR (CDCl_3_, 600 MHz) δ 2.34 (s, 3H), 2.75 (s, 4H), 3.76 (s, 3H), 3.80 (s, 3H), 3.84 (s, 3H), 3.85 (s, 3H), 6.69 (s, 1H), 6.72–6.74 (d, *J* = 8.7 Hz, 2H), 6.77–6.78 (d, *J* = 8.1 Hz, 1H), 6.81 (d, *J* = 8.9 Hz, 1H); ^13^C NMR (CDCl_3_, 150 MHz) δ 30.1, 32.4, 36.1, 55.9, 56.1, 56.1, 109.1, 111.3, 111.4, 112.1, 120.5, 128.0, 133.0, 135.2, 147.3, 148.8, 149.8, 151.1, 205.5; HRMS (ESI): [M + H]^+^ calcd for C_20_H_25_O_5_, 345.1702; found 345.1697; anal. calcd for C_20_H_24_O_5_: C, 69.75; H, 7.02; found: C, 69.56; H, 6.73.

**General procedure for the synthesis of phenols 9–11 by hydrogenolytic cleavage of benzyl ether protecting groups**: An autoclave was equipped with a stirring bar and charged with O-protected dihydrostilbenes **8a**,**b** (0.8 mmol), palladium on charcoal (50 mg) and ethyl acetate (12 mL). The autoclave was connected to the hydrogen source and rinsed carefully with hydrogen. Thereafter, the mixture was stirred at 25 °C and 3 bar hydrogen pressure for 1–3 d. Then, the mixture was filtered through a frit filled with silica gel that was rinsed five times with ethyl acetate. The combined filtrates were concentrated under reduced pressure and the crude product was purified by column chromatography using mixtures of ethyl acetate and *n*-hexane. According to this procedure, the following compounds were obtained:

**1-[2-(3,4-Dimethoxyphenethyl)-3,6-dihydroxyphenyl)ethan-1-one (9):** Prepared from **8a** (741 mg, 1.5 mmol), palladium on charcoal (200 mg) in ethyl acetate (15 mL) at 3 bar hydrogen pressure for 72 h. The crude product was purified by column chromatography (silica gel; ethyl acetate/*n*-hexane, 1:7) to give **9** as a greenish syrup in 75% yield (356 mg); *R*_f_ 0.1; ^1^H NMR (CDCl_3_, 600 MHz) δ 2.63 (s, 3H), 2.87 (dd, *J* = 9.0, 6.8 Hz, 2H), 3.09 (dd, *J* = 9.0, 6.8 Hz, 2H), 3.83 (s, 3H), 3.85 (s, 3H), 4.41 (br s, 1H), 6.64 (d, *J* = 1.9 Hz, 1H), 6.72–6.75 (m, 2H), 6.79 (d, *J* = 8.00 Hz, 1H), 6.83 (d, *J* = 8.9 Hz, 1H), 9.35 (br s, 1H); ^13^C NMR (CDCl_3_, 150 MHz) δ 30.6, 32.7, 36.1, 56.0, 56.1, 111.5, 111.9, 116.3, 120.3, 121.8, 125.2, 127.5, 134.1, 147.0, 147.7, 149.1, 152.9, 205.9; HRMS (ESI): [M + H]^+^ calcd for C_18_H_21_O_5_, 317.1389; found, 317.1384.

**1-[3,6-Dihydroxy-2-(4-methoxyphenethyl)phenyl]ethan-1-one (10):** Prepared from **8b** (510 mg, 1.1 mmol), palladium on charcoal (135 mg) in ethyl acetate (15 mL) at 3 bar hydrogen pressure for 25 h. The crude product was purified by column chromatography (silica gel; ethyl acetate/*n*-hexane, 1:7) to give **10** (204 mg, 65%) as a greenish-brownish syrup; *R*_f_ 0.12; ^1^H NMR (CDCl_3_, 600 MHz) δ 2.64 (s, 3H), 2.85–2.87 (m, 2H), 3.06–3.09 (m, 2H), 3.79 (s, 3H), 4.45 (br s, 1H), 6.73 (d, *J* = 6.73 Hz, 1H), 6.83–6.85 (m, 3H), 7.10 (d, *J* = 7.1 Hz, 2H); ^13^C NMR (CDCl_3_, 150 MHz) δ 30.8, 32.7, 35.6, 55.5, 114.2, 114.2, 116.3, 121.8, 125.0, 127.6, 129.4, 129.4, 133.6, 146.0, 153.1, 158.3, 206.0; HRMS (ESI): [M + H]^+^ calcd for C_17_H_19_O_4_, 287.1283; found, 287.1279.

A forerun in the column chromatography contained mono-deprotected 1-[3-(benzyloxy)-6-hydroxy-2-(4-methoxyphenethyl)phenyl]ethan-1-one (**11**) that was still contaminated with a small amount of hydroquinone **10**. Yield: 68 mg (16%); *R*_f_ 0.3; ^1^H NMR (CDCl_3_, 600 MHz) δ 2.65 (s, 3H), 2.82–2.85 (m, 2H), 3.04–3.09 (m, 2H), 3.78 (s, 3H), 5.02 (s, 2H), 6.81–6.83 (m, 3H), 6.99 (d, *J* = 8.2 Hz, 2H), 7.13–7.16 (m, 3H), 7.35–7.37 (m, 1H), 7.44 (d, 2H), 9.31 (br s, 1H); ^13^C NMR (CDCl_3_, 150 MHz) δ 32.7, 35.4, 35.8, 55.4, 71.6, 114.0, 114.0, 115.9, 118.7, 118.7, 121.4, 126.8, 127.6, 128.2, 128.8, 129.4, 129.8, 130.5, 134.0, 137.5, 152.5, 154.6, 158.1, 205.2; ESIMS *m*/*z* (%): 377.2 [M + 1] (100); HRMS (ESI): [M + H]^+^ calcd for C_24_H_25_O_4_, 377.1753; found, 377.1748.

**(2*R*,3*R*,4*S*,5*R*,6*R*)-2-(Acetoxymethyl)-6-[3-acetyl-2-(3,4-dimethoxyphenethyl)-4-hydroxyphenoxy]tetrahydro-2*H*-pyran-3,4,5-triyl triacetate (12):** A 50 mL two-necked flask was equipped with a stirring bar and a connection to the combined nitrogen/vacuum line. The flask was charged with dihydrostilbene **9** (330 mg, 1.04 mmol) and β-glucose pentaacetate (814 mg, 2.08 mmol) and closed with a septum. The air in the flask was replaced by nitrogen. Then, dichloromethane (4 mL) and boron trifluoride etherate (0.4 mL, 3.12 mmol) were injected by syringe. The mixture was stirred at 25 °C for 2 d. Thereafter, saturated aqueous sodium hydrogen carbonate (10 mL) was added and the mixture was extracted five times with 15 mL portions of dichloromethane. The combined organic layers were washed with brine and dried with magnesium sulfate. The solvent was removed in a rotary evaporator and the crude product (1.08 g) was purified by column chromatography (silica gel; ethyl acetate/*n*-hexane, 1:4 to 1:2) to give 91 mg (14%) of pure glycoside **12** as a colorless syrup. ^1^H NMR (CDCl_3_, 600 MHz) δ 2.04 (s, 3H), 2.06 (s, 3H), 2.07 (s, 3H), 2.08 (s, 3H), 2.64 (s, 3H), 2.77–2.82 (m, 1H), 2.92–2.97 (m, 1H), 3.08–3.14 (m, 2H), 3.85 (s, 3H), 3.87 (s, 3H), 4.0–4.14 (m, 2H), 4.26 (dd, *J* = 12.4, 4.7 Hz, 1H), 5.10 (ddd, *J* = 10.3, 3.8, 1.1 Hz, 1H), 5.18 (t, *J* = 5.2 Hz, 1H), 5.59 (d, *J* = 3.5 Hz, 1H), 5.75 (t, *J* = 10.2 Hz, 1H), 6.71 (s, 1H), 6.78 (dd, *J* = 9.0, 1.1 Hz, 1H), 6.82–6.85 (m, 2H), 7.28 (dd, *J* = 9.1, 1.0 Hz, 1H), 8.99 (brs, 1H); ^13^C NMR (CDCl_3_, 150 MHz) δ 20.7, 20.8, 20.8, 20.9, 31.0, 32.7, 36.6, 56.0, 56.1, 60.6, 61.8, 68.2, 68.4, 70.2, 70.4, 95.8, 111.7, 116.2, 120.5, 120.6, 120.8, 124.7, 134.0, 147.7, 149.1, 170.1, 170.7, 205.6, 206.1, 206.2, 210.7, 213.0; HRMS: [M + H]^+^ calcd for C_32_H_38_NaO_14_, 669.2159; found, 669.2154.

***epi*-Scorzodihydrostilbene D (13):** A mixture of **12** (18 mg, 0.03 mmol), methanol (1 mL) and sodium ethoxide (3 mg, 0.06 mmol) was stirred at 25 °C for 4 h. The mixture was filtered and the filtrate was concentrated under reduced pressure. The residue was purified by semi-preparative reversed-phase HPLC (methanol–water gradient) to give 11 mg (17%) of pure *epi*-scorzodihydrostilbene D (**13**) as a colorless syrup. ^1^H NMR (CD_3_OD, 600 MHz) δ 2.33 (s, 3H), 2.69–2.99 (m, 4H), 3.45 (t, *J* = 8.9 Hz, 1H), 3.63 (dd, *J* = 9.6 Hz, 3.3 Hz, 1H), 3.72 (m, 2H), 3.77–3.83 (m, 7H), 3.95 (t, *J* = 9.3 Hz, 1H), 5.40 (s, 1H), 6.67 (d, *J* = 8.9 Hz, 1H), 6.76 (d, *J* = 8.0 Hz, 1H), 6.81 (s, 1H), 6.84 (d, *J* = 7.9 Hz, 1H), 7.21 (d, *J* = 8.8 Hz, 1H); ^13^C NMR (CD_3_OD, 150 MHz) δ 31.3, 32.4, 37.3, 56.5, 56.6, 62.5, 68.5, 71.7, 73.4, 74.5, 75.2, 100.2, 113.2, 113.9, 114.7, 118.6, 121.9, 130.2, 131.8, 136.9, 148.6, 150.1, 150.2, 208.1; HRMS: [M + H]^+^ calcd for C_24_H_30_NaO_10_, 501.1737; found, 501.1724.

## Supporting Information

File 1 ^1^H NMR and ^13^C NMR spectra of all new compounds.

## References

[1] Xiao K, Zhang H-J, Xuan L-J, Zhang J, Xu Y-M, Bai D-L, Atta-ur-Rahman (2008). Stilbenoids: Chemistry and bioactivities. Studies in Natural Products Chemistry.

[2] Grubov V I (1982). Key to the Vascular Plants of Mongolia.

[3] Gubanov I A (1996). The conspectus of flora outer Mongolia (vascular plants).

[4] Ligaa U (1996). Medicinal Plants from Mongolia Used in Mongolian Traditional Medicine.

[5] Wang Y, Edrada-Ebel R, Tsevegsuren N, Sendker J, Braun M, Wray V, Lin W, Proksch P (2009). J Nat Prod.

[6] Arockiam P B, Bruneau C, Dixneuf P H (2012). Chem Rev.

[7] Venkateswarlu S, Raju M S S, Subbaraju G V (2002). Biosci, Biotechnol, Biochem.

[8] Zhang W-G, Zhao R, Ren J, Ren L-X, Lin J-G, Liu D-L, Wu Y-L, Yao X-S (2007). Arch Pharm (Weinheim, Ger).

[9] Moodie L W K, Trepos R, Cervin G, Bråthen K A, Lindgård B, Reiersen R, Cahill P, Pavia H, Hellio C, Svenson J (2017). J Nat Prod.

[10] Weimann K (2014). Erste Synthese eines Scorzodihydrostilben-Aglycons und Glycosylierung zu Epi-Scorzodihydrostilben D.

[11] Murai S, Kakiuchi F, Sekine S, Tanaka Y, Kamatani A, Sonoda M, Chatani N (1993). Nature.

[12] Murai S, Kakiuchi F, Sekine S, Tanaka Y, Kamatani A, Sonoda M, Chatani N (1994). Pure Appl Chem.

[13] Kakiuchi F, Sekine S, Tanaka Y, Kamatani A, Sonoda M, Chatani N, Murai S (1995). Bull Chem Soc Jpn.

[14] Murai S, Chatani N, Kakiuchi F (1997). Pure Appl Chem.

[15] Lewis L N, Smith J F (1986). J Am Chem Soc.

[16] Martinez R, Genet J-P, Darses S (2008). Chem Commun.

[17] Greene T W, Wuts P G M (1991). Protective Groups in Organic Synthesis.

[18] Augustine R L (1965). Catalytic Hydrogenation.

[19] Helferich B, Schmitz-Hillebrecht E (1933). Ber Dtsch Chem Ges B.

[20] Cocinero E J, Gamblin D P, Davis B G, Simons J P (2009). J Am Chem Soc.

[21] Giles R G F, Joll C A (1999). J Chem Soc, Perkin Trans 1.

[22] Sun B, Hoshino J, Jermihov K, Marler L, Pezzuto J M, Mesecar A D, Cushman M (2010). Bioorg Med Chem.

