# Supplementary material for: Synthesis of the aglycon of scorzodihydrostilbenes B and D
Source: Beilstein J Org Chem. 2019 Mar 6;15:610–6. doi: 10.3762/bjoc.15.56 (PMC6423597; doi:10.3762/bjoc.15.56)
Supplement: File 1 — 1H NMR and 13C NMR spectra of all new compounds. [file Beilstein_J_Org_Chem-15-610-s001.pdf]

BEILSTEIN JOURNAL OF ORGANIC CHEMISTRY

Supporting Information

for

Synthesis of the aglycon of scorzodihydrostilbenes B and D

Katja Weimann and Manfred Braun

Beilstein J. Org. Chem. **2019,** *15,* 610–616. doi:10.3762/bjoc.15.56

^{1}H NMR and ^{13}C NMR spectra of all new compounds

License and Terms: This is a supporting information file under the terms of the Creative Commons Attribution License (http://creativecommons.org/licenses/by/4.0). Please note that the reuse, redistribution and reproduction in particular requires that the authors and source are credited.
The license is subject to the *Beilstein Journal of Organic Chemistry* terms and conditions: (https://www.beilstein-journals.org/bjoc)

1-[3,6-Bis(benzyloxy)-2-(3,4-dimethoxyphenethyl)phenyl)]ethan-1-one (**8a**)

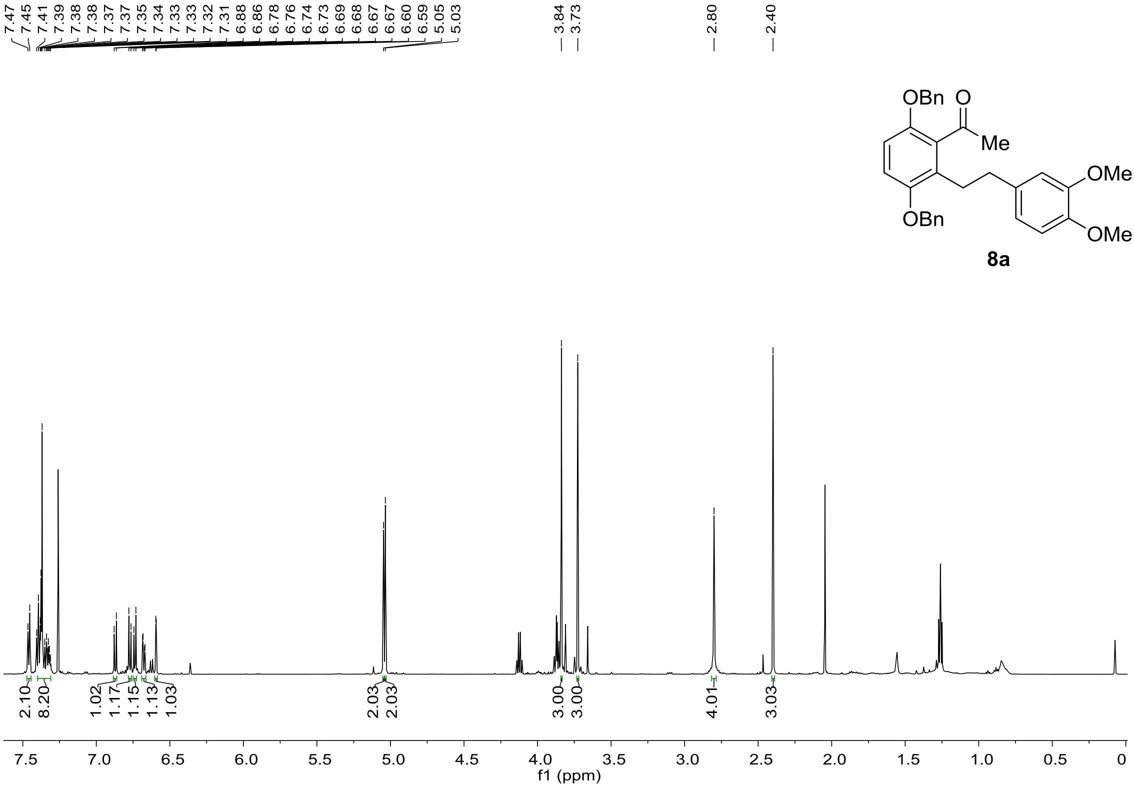

^{1}H NMR of **8a** in $CDCl_3$ at 298 K.

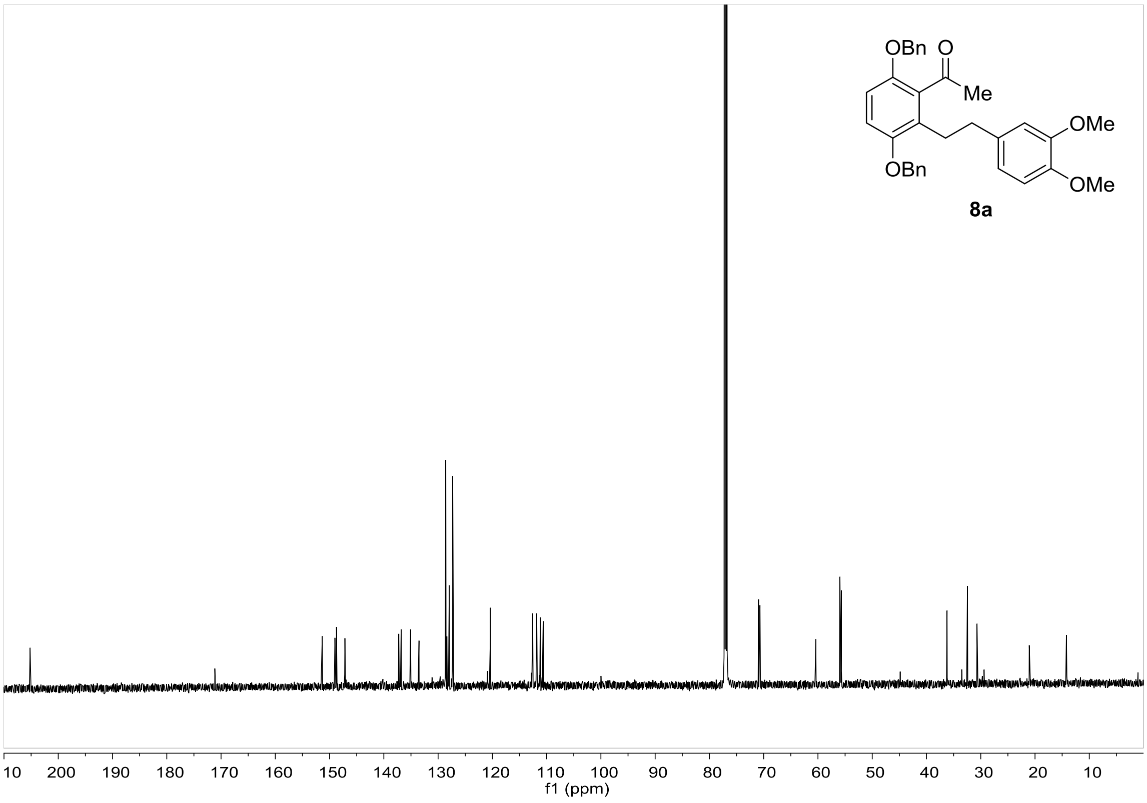

^{13}C NMR of **8a** in $CDCl_3$ at 298 K.

1-[3,6-Bis(benzyloxy)-2-(4methoxyphenethyl)phenyl]ethan-1-one (**8b**)

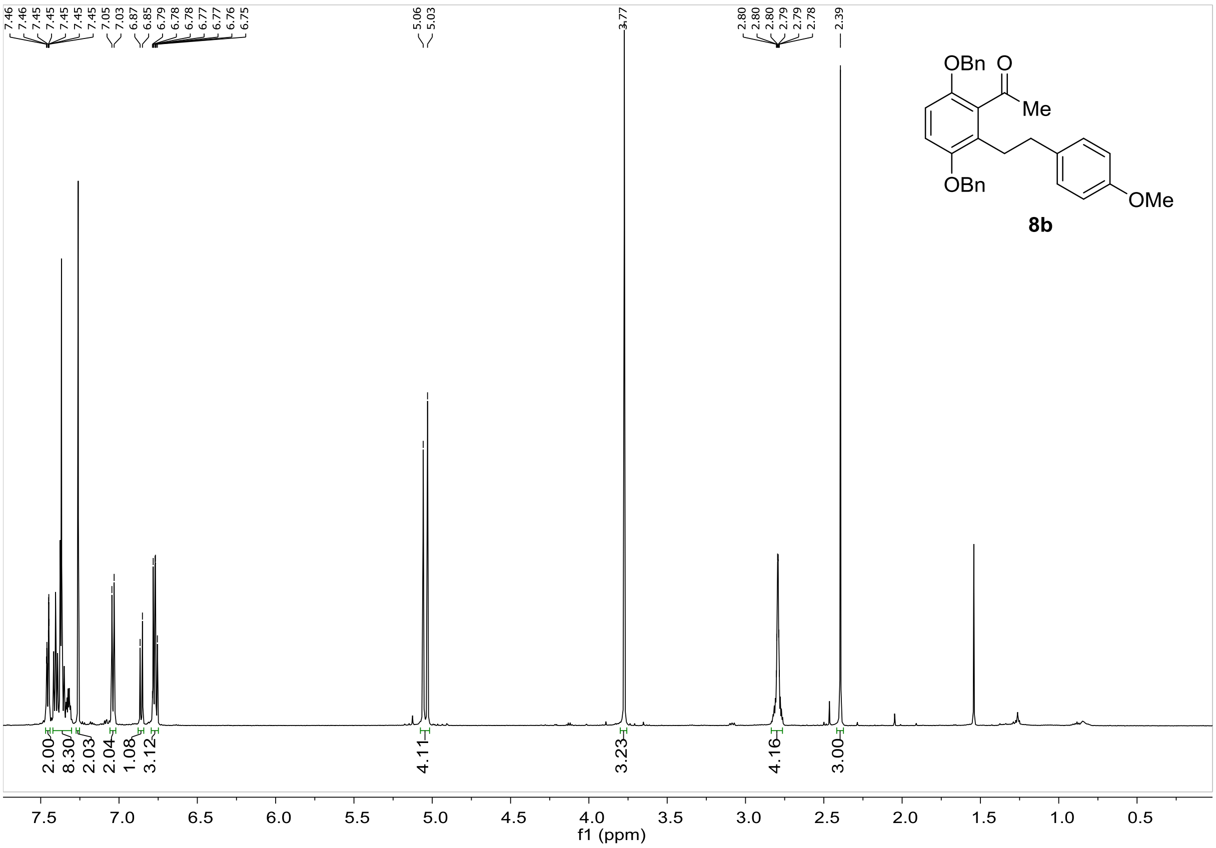

^{1}H NMR of **8b** in $CDCl_3$ at 298 K.

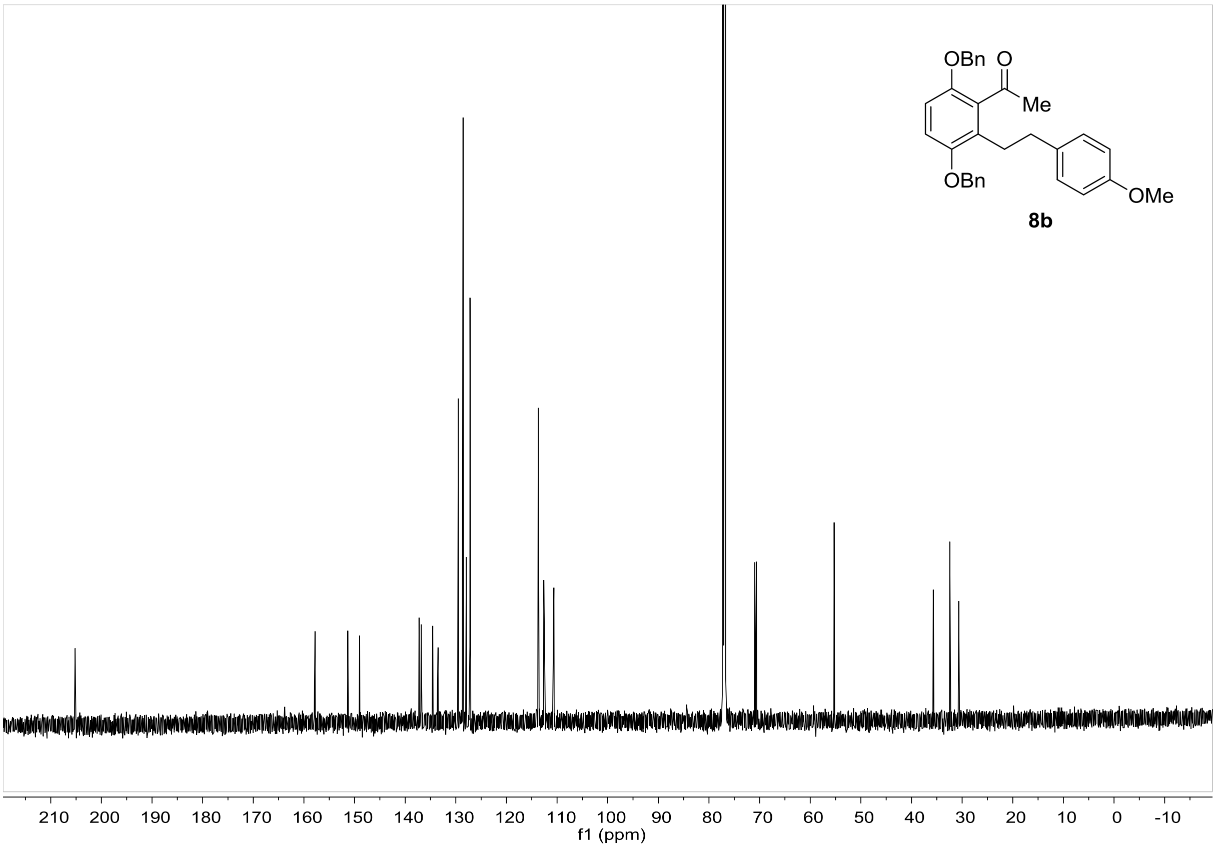

^{13}C NMR of **8b** in $CDCl_3$ at 298 K.

1-[3-(Benzyloxy)-6-methoxy-2-(4-methoxyphenethyl)phenyl]ethan-1-one (**8c**)

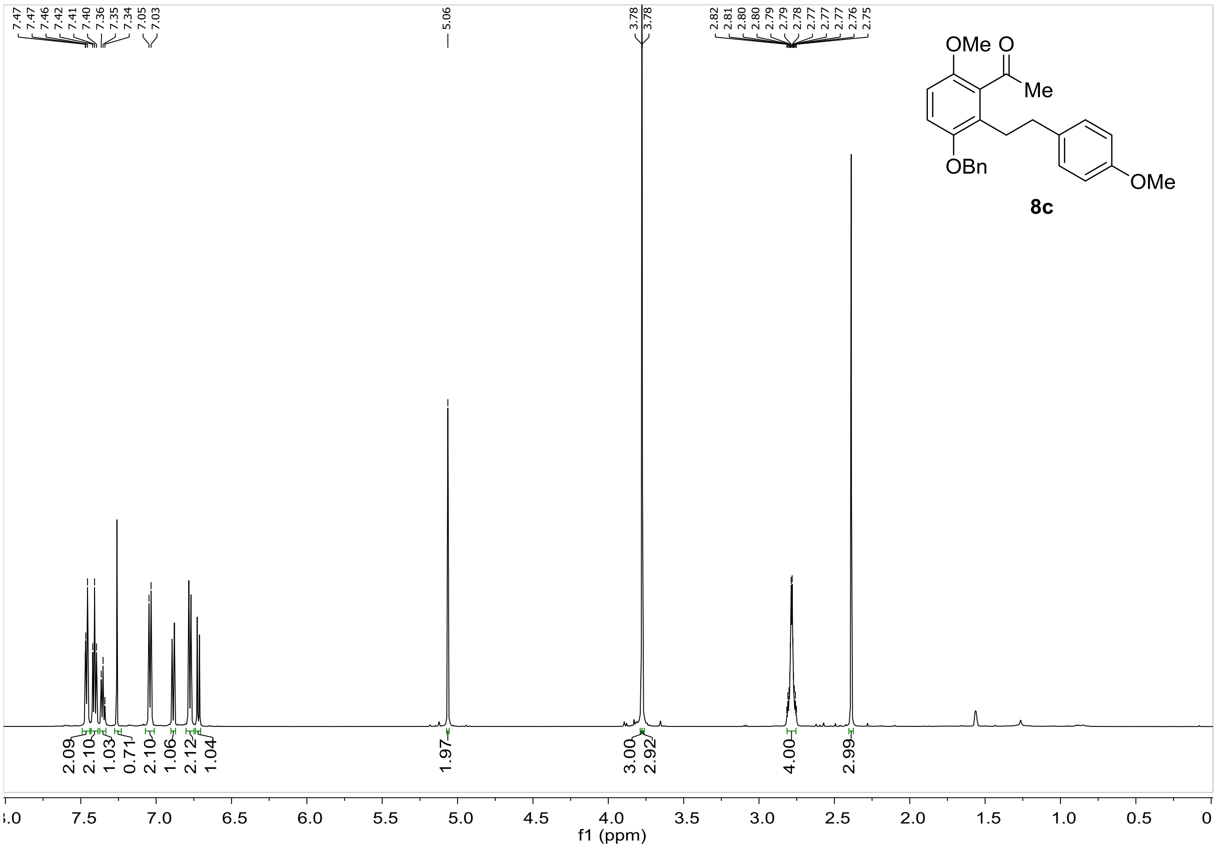

^{1}H NMR of **8c** in $CDCl_3$ at 298 K.

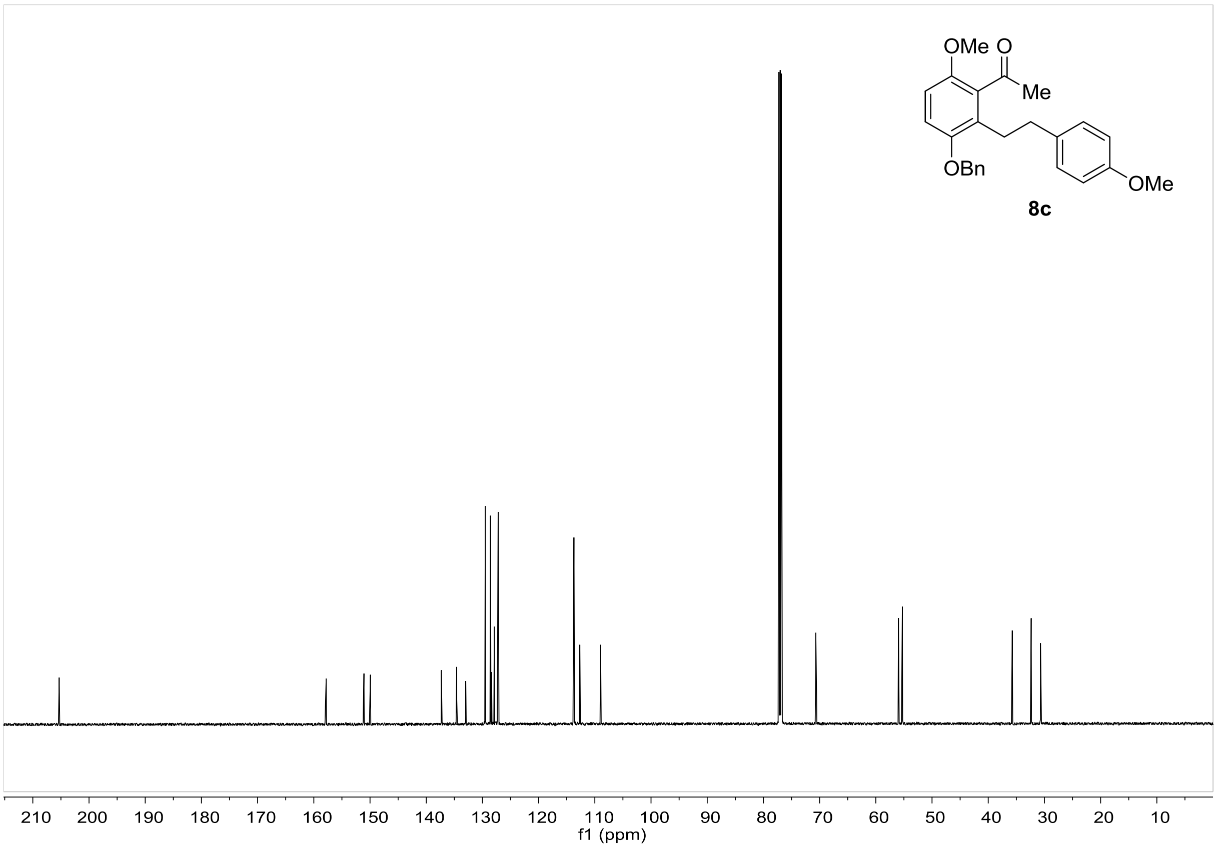

^{13}C NMR of **8c** in $CDCl_3$ at 298 K.

1-[2-(3,4-Dimethoxyphenethyl)-3,6-dimethoxyphenyl]ethan-1-one (**8d**)

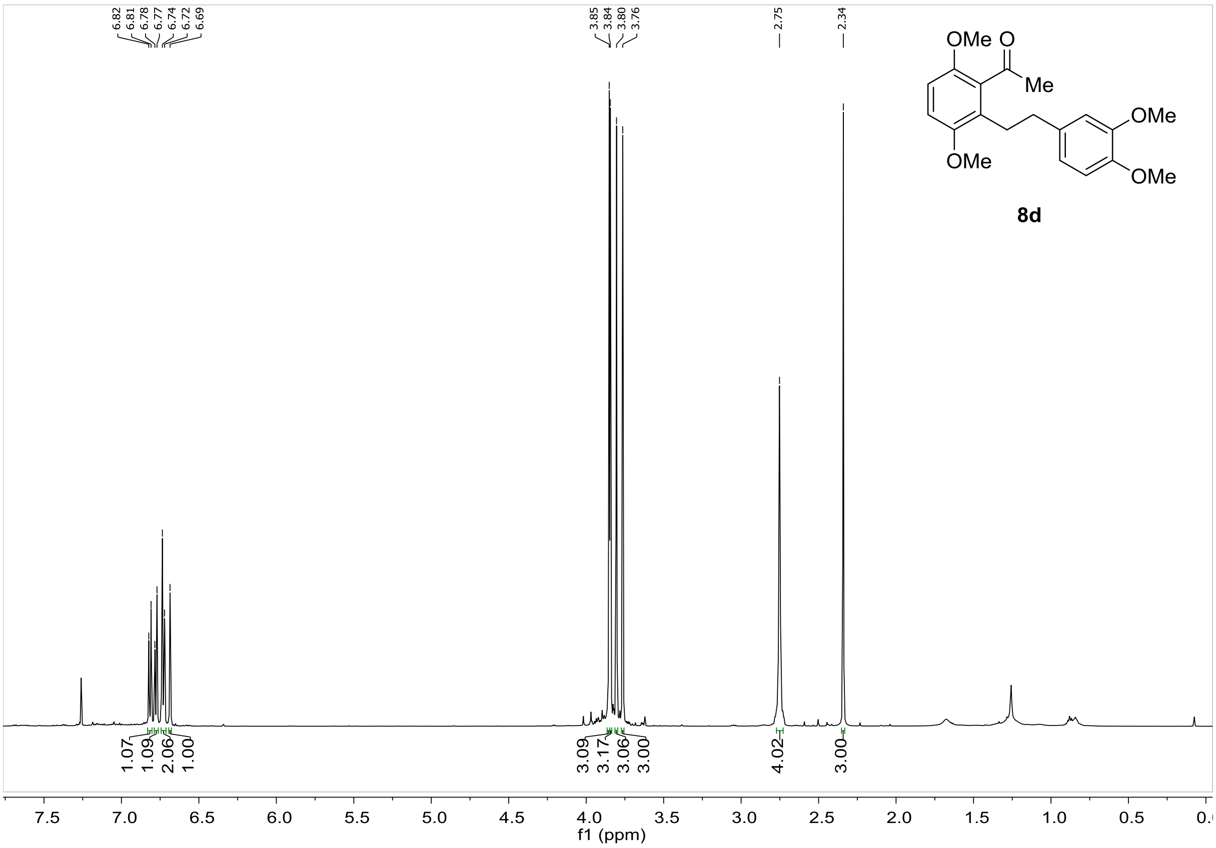

^{1}H NMR of **8d** in $CDCl_3$ at 298 K.

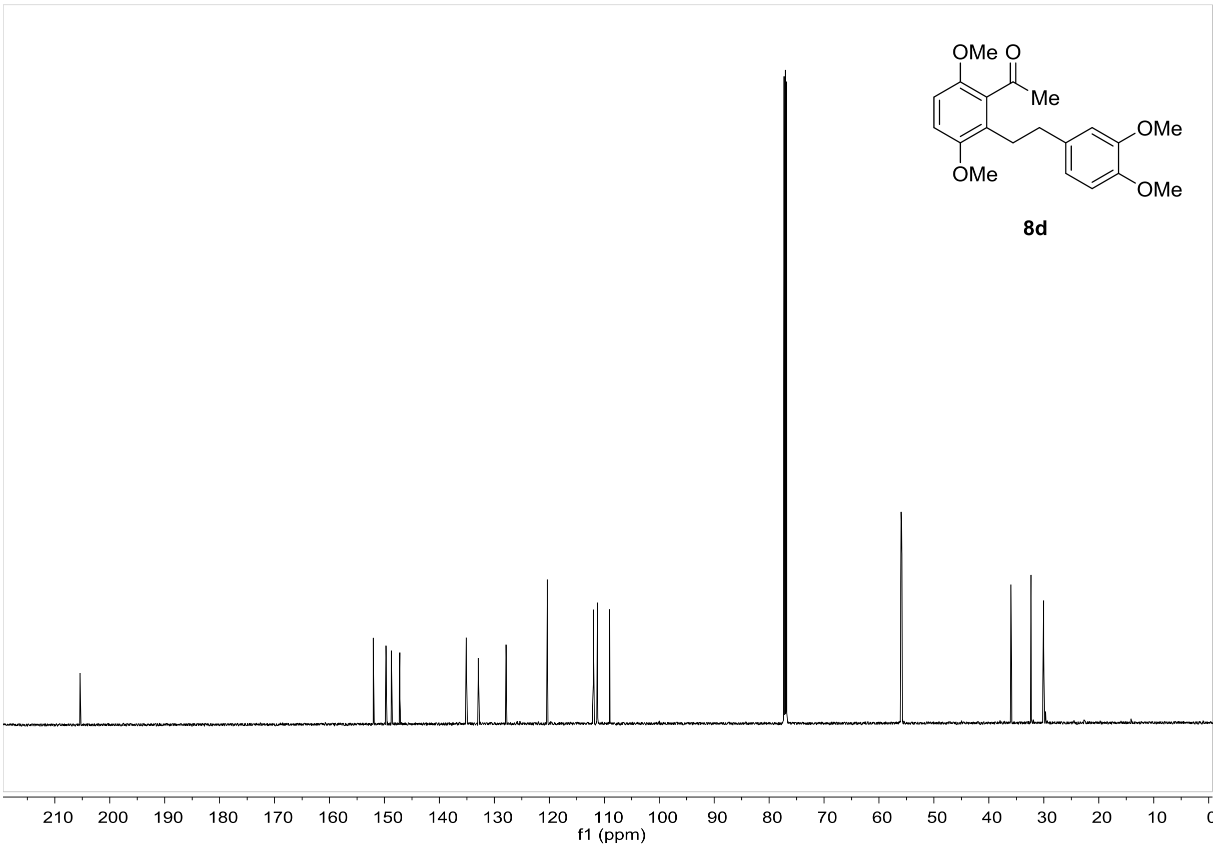

^{13}C NMR of **8d** in $CDCl_3$ at 298 K.

1-[2-(3,4-Dimethoxyphenethyl)-3,6-dihydroxyphenyl)ethan-1-one (**9**)

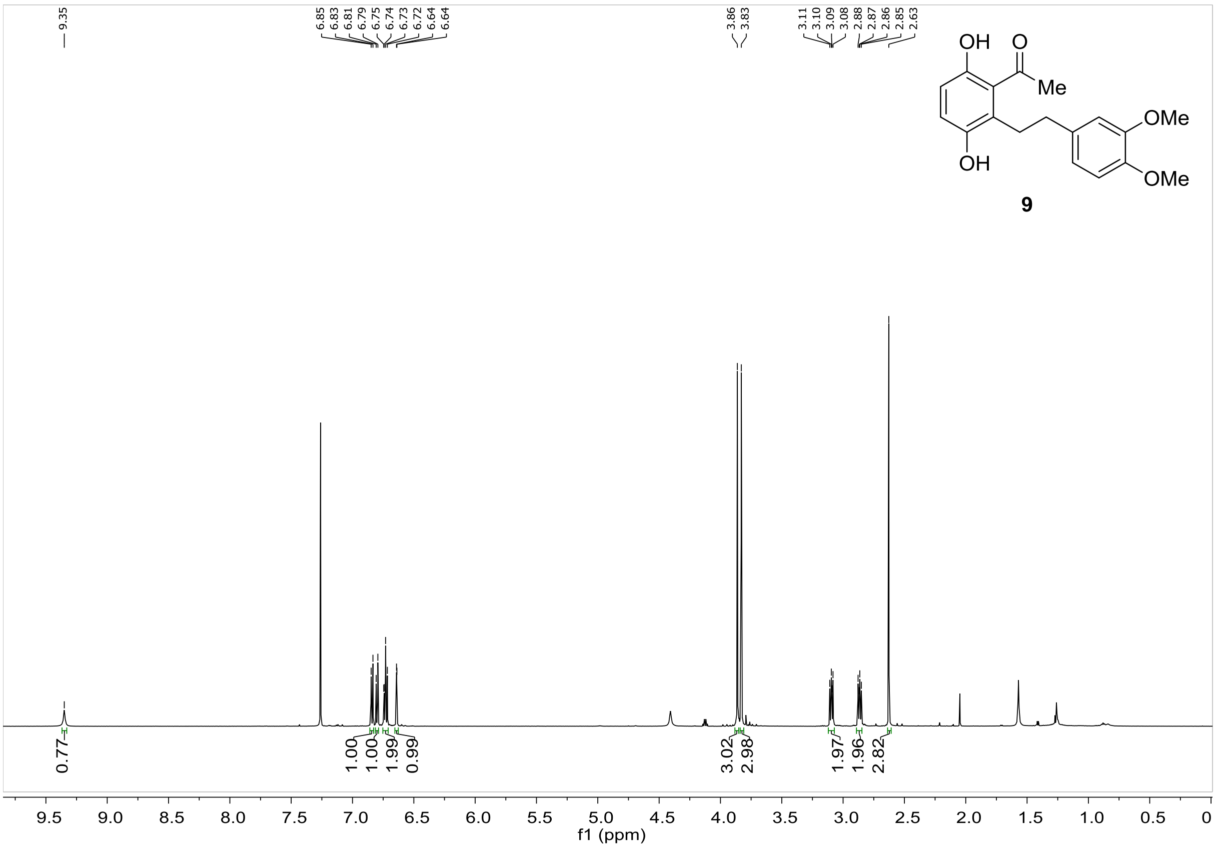

^{1}H NMR of **9** in $CDCl_3$ at 298 K.

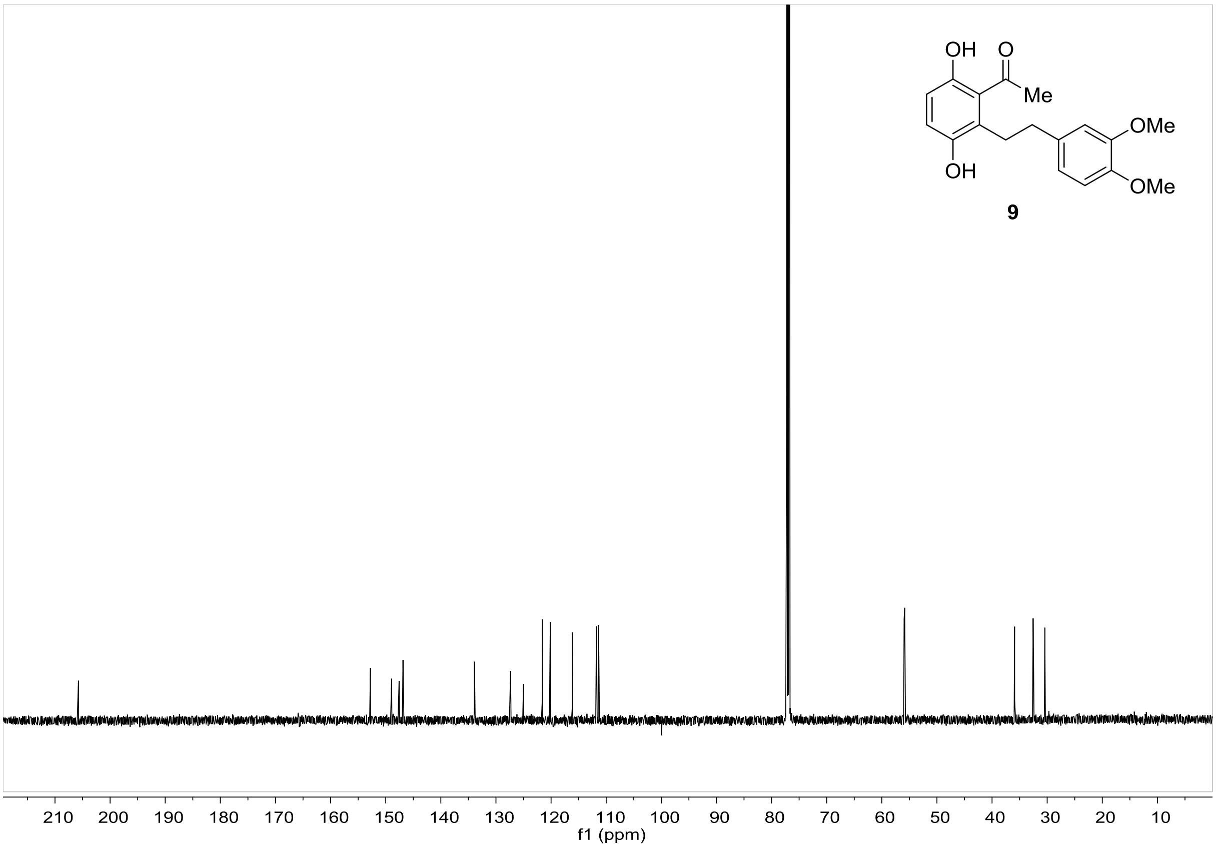

^{13}C NMR of **9** in $CDCl_3$ at 298 K.

1-[3,6-Dihydroxy-2-(4-methoxyphenethyl)phenyl]ethan-1-one (**10**)

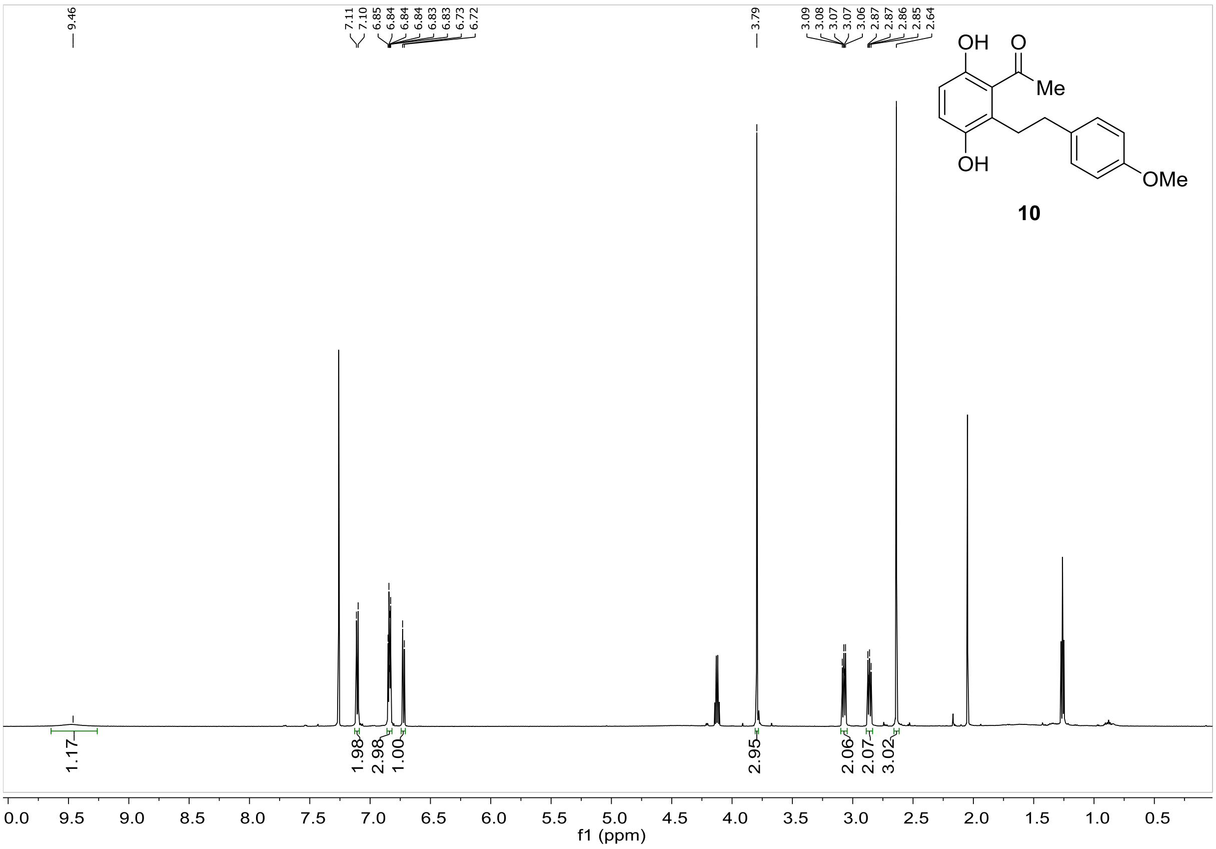

^{1}H NMR of **10** in $CDCl_3$ at 298 K.

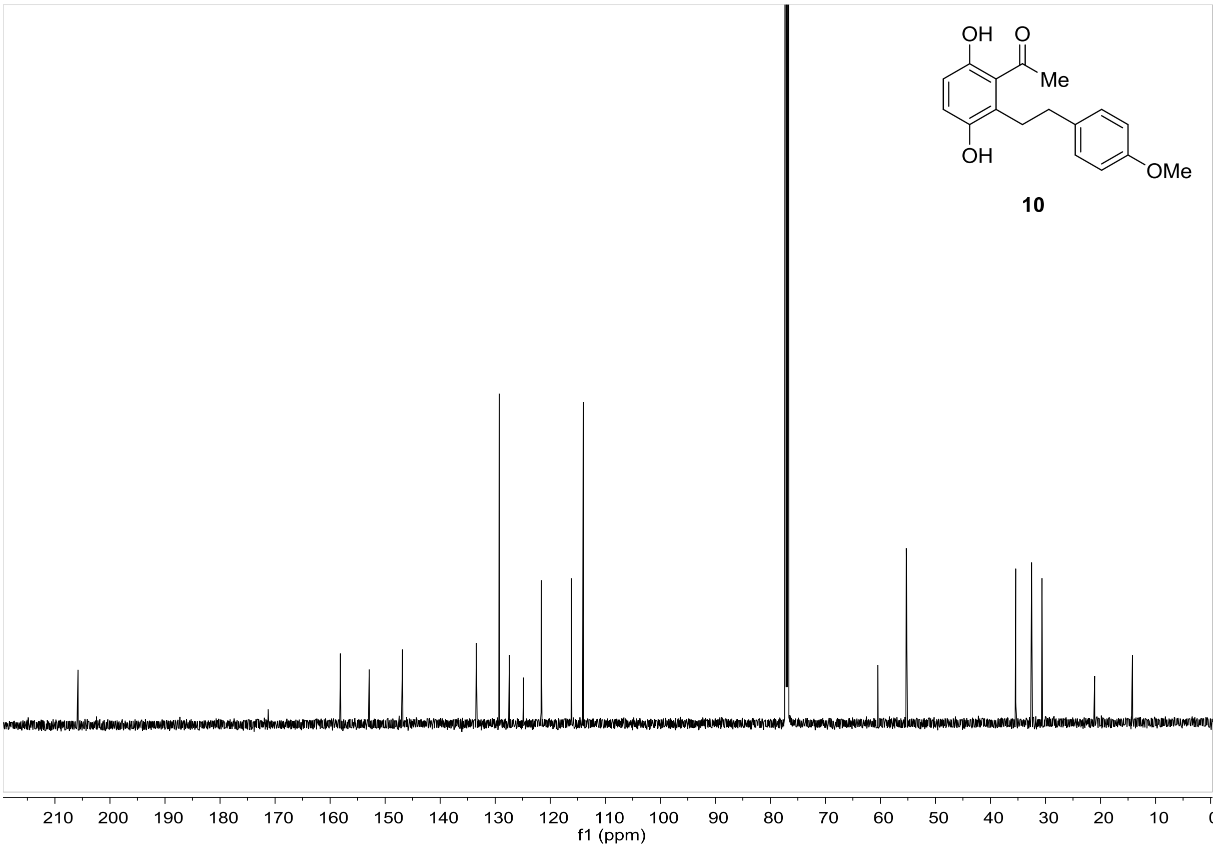

^{13}C NMR of **10** in $CDCl_3$ at 298 K.

1-[3-(Benzyloxy)-6-hydroxy-2-(4methoxyphenethyl)phenyl]ethan-1-one (**11**) and 1-[3,6-Dihydroxy-2-(4-methoxyphenethyl)phenyl]ethan-1-one (**10**) as contamination.

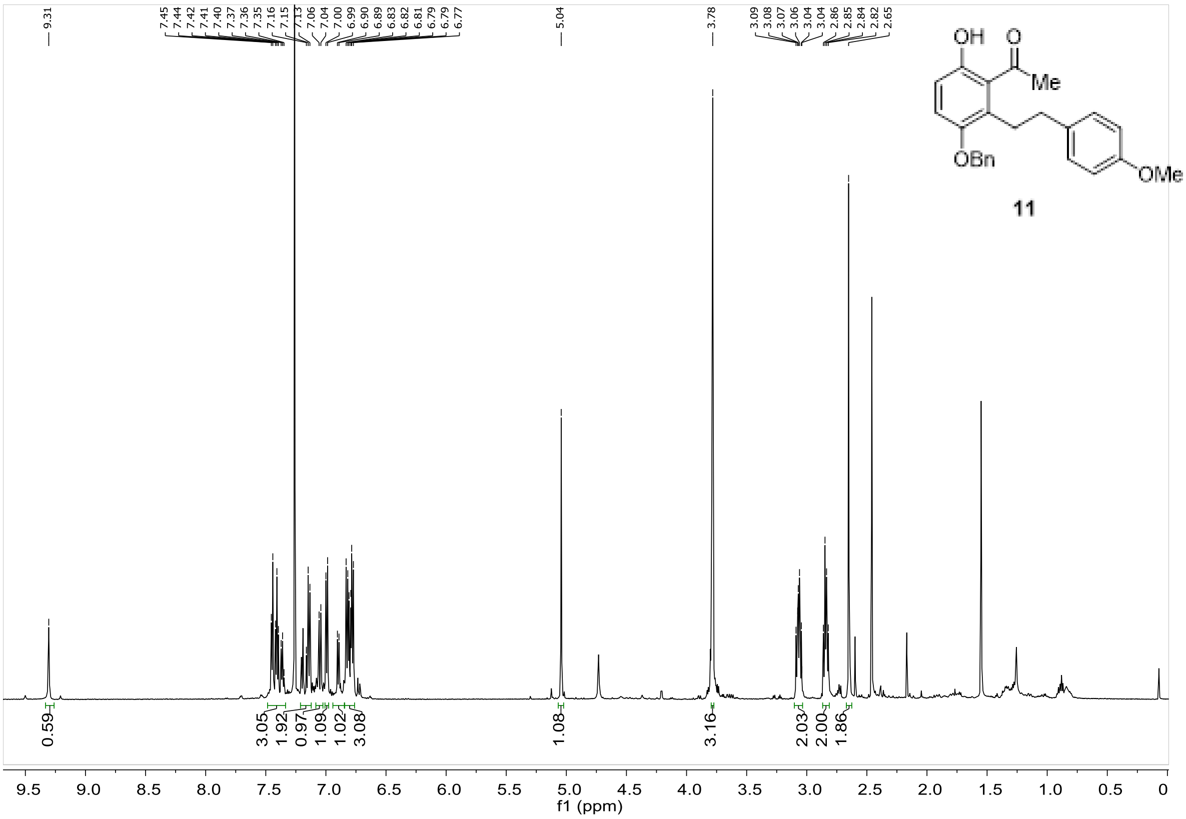

^{1}H NMR of a fraction including **11** in $CDCl_3$ at 298 K.

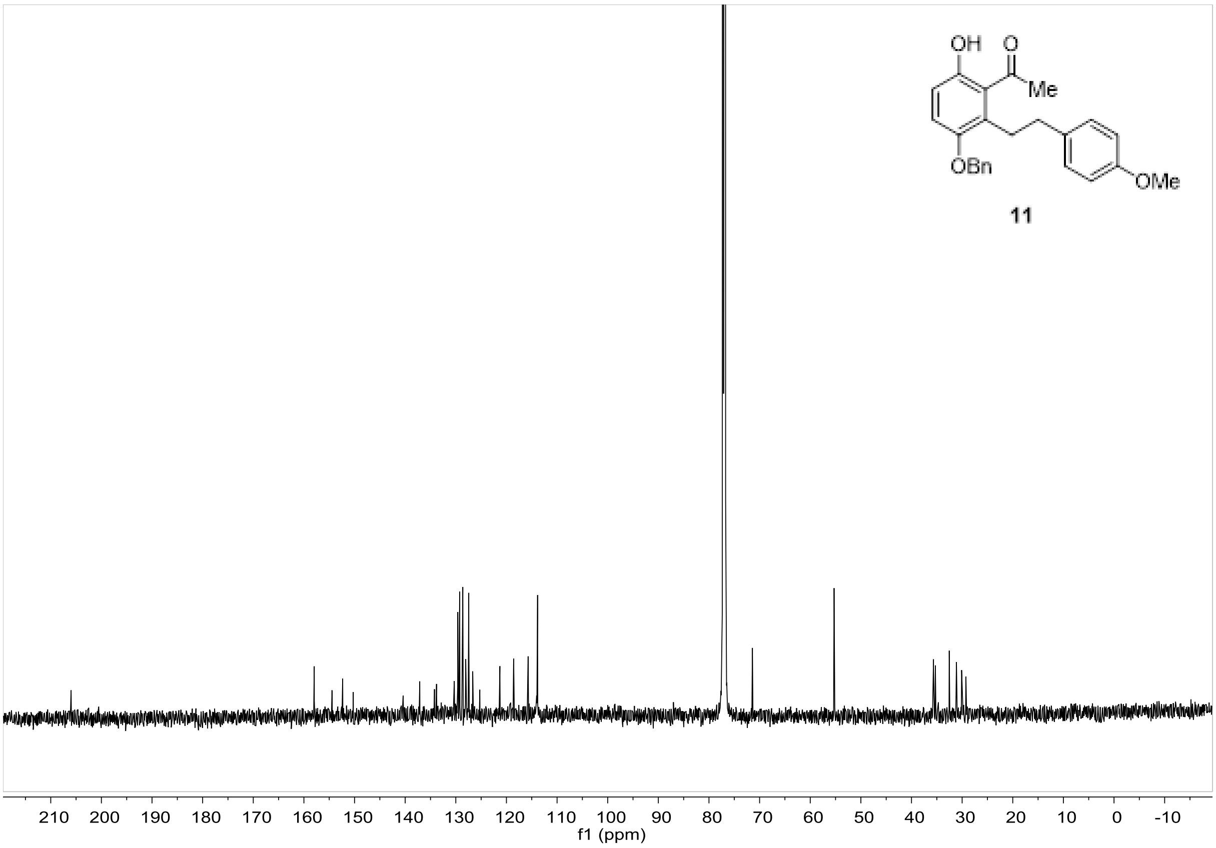

^{13}C NMR of a fraction including **11** in $CDCl_3$ at 298 K.

(2*R*,3*R*,4*S*,5*R*,6*R*)-2-(Acetoxymethyl)-6-([3-acetyl-2-(3,4-dimethoxyphenethyl)-4-hydroxyphenoxy]tetrahydro-2H-pyran-3,4,5-triyl triacetate (**12**)

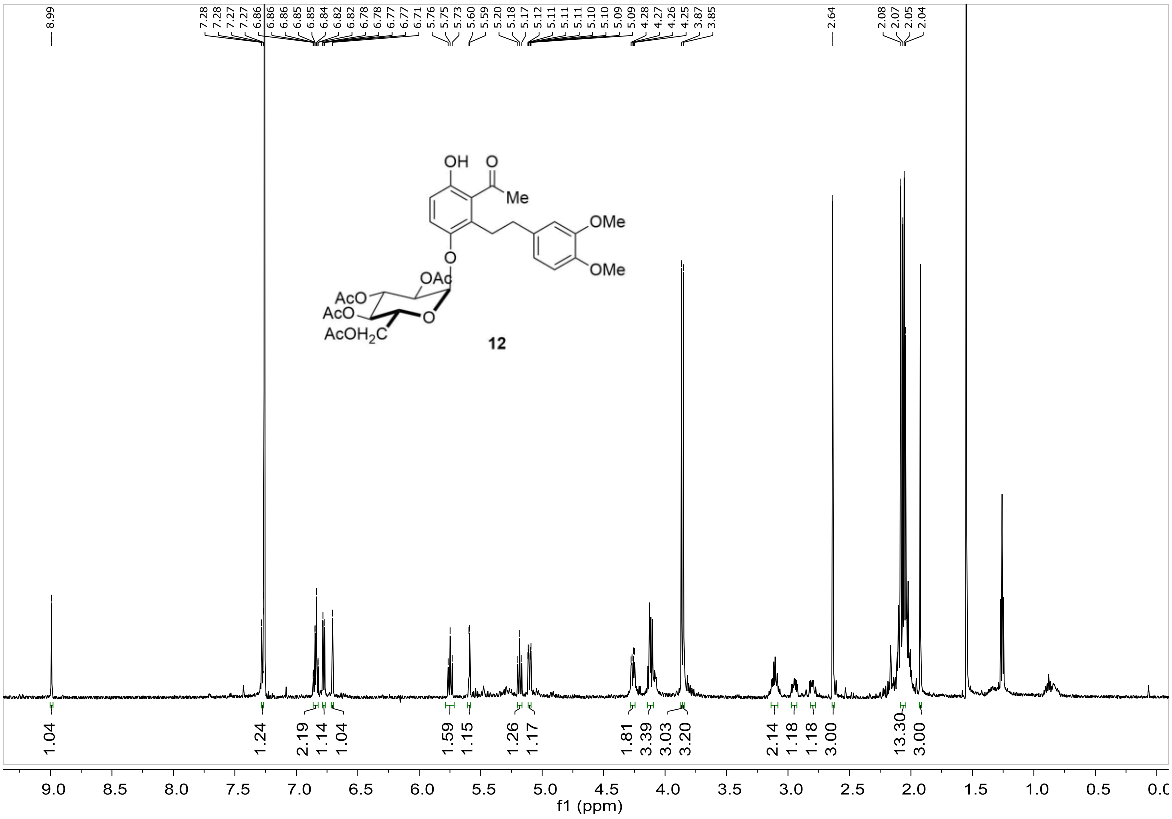

^{1}H NMR of **12** in $CDCl_3$ at 298 K.

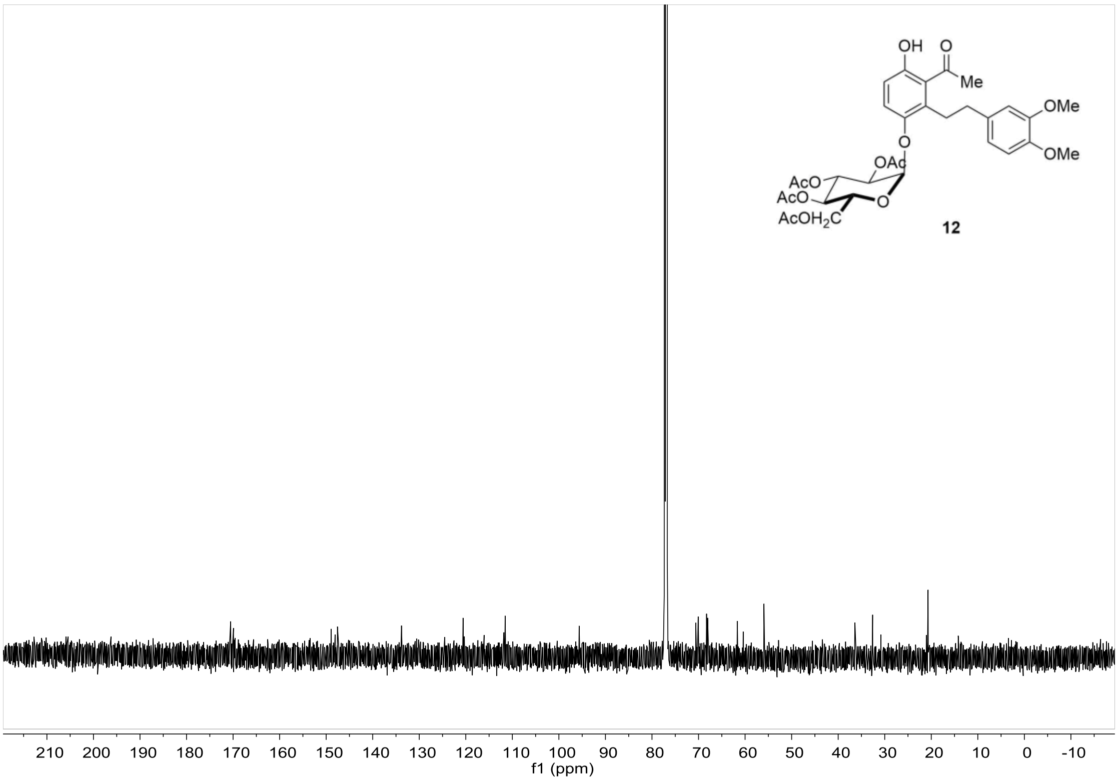

^{13}C NMR of **12** in $CDCl_3$ at 298 K.

epi-Scorzodihydrostilbene D (**13**)

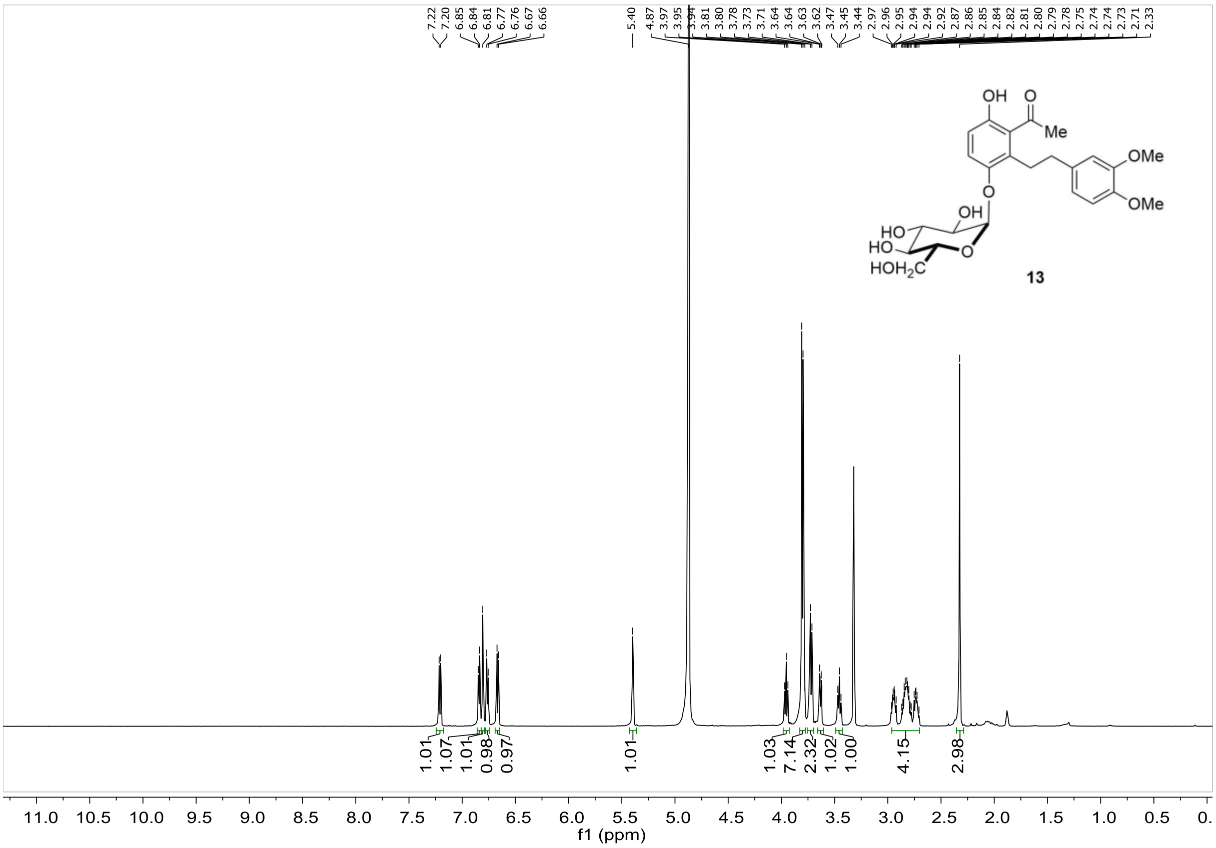

^{1}H NMR of **13** in CD_3OD at 298 K.

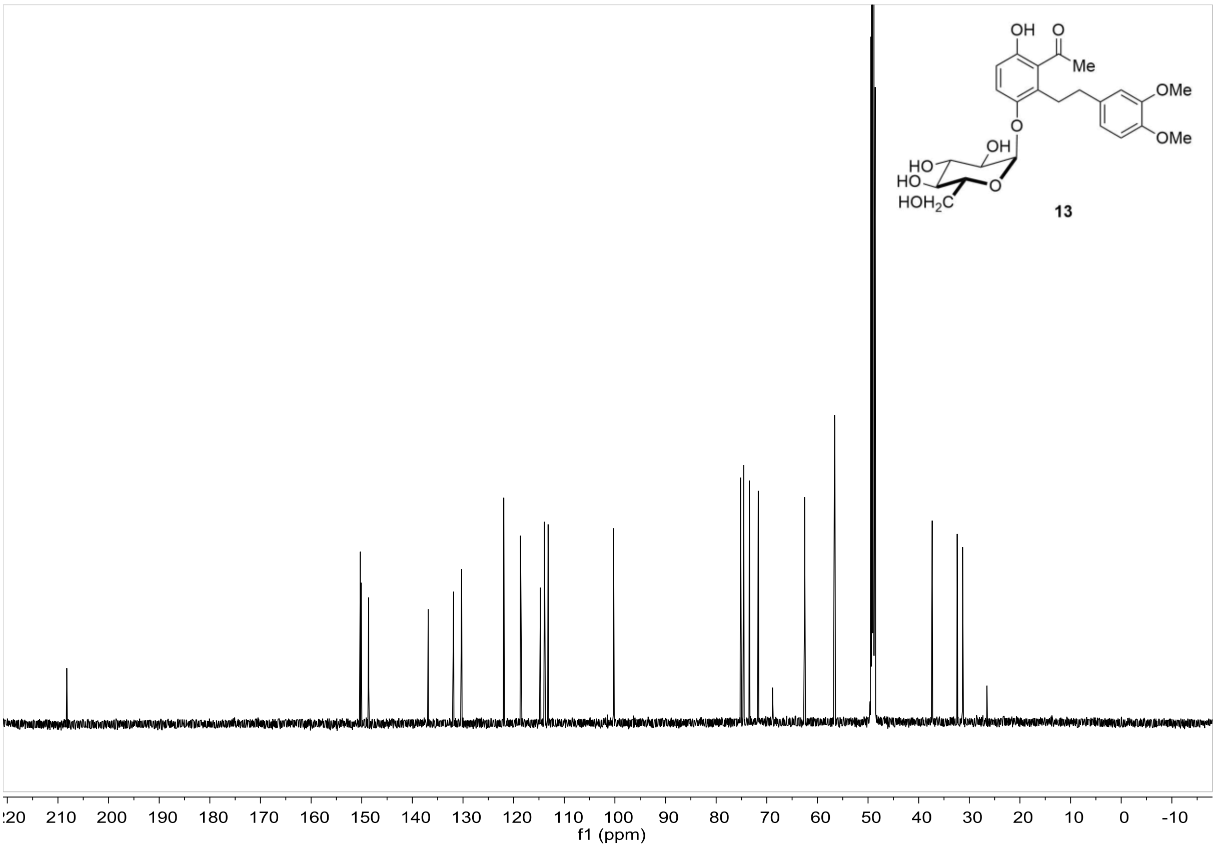

^{13}C NMR of **13** in CD_3OD at 298 K.
